# Does Programmed Cell Death 1 Ligand (Pd-L1) Expression Predict Recurrence in Women With Endometrioma?

**DOI:** 10.7759/cureus.56262

**Published:** 2024-03-16

**Authors:** Hale Goksever Celik, Engin Celik, Mehmet Uhri, Ercan Bastu, Mete Gungor, Faruk Buyru

**Affiliations:** 1 Obstetrics and Gynecology, Acibadem University, Istanbul, TUR; 2 Obsterics and Gynecology, Acibadem Atasehir Hospital, Istanbul, TUR; 3 Pathology, Saglik Bilimleri University Istanbul Bakırkoy Dr. Sadi Konuk Training and Research Hospital, Istanbul, TUR; 4 Obstetrics and Gynecology, Biruni University, Istanbul, TUR; 5 Obstetrics and Gynecology, Acibadem Fulya Hospital, Istanbul, TUR

**Keywords:** ca125, pd-l1 expression, recurrence, endometrioma, endometriosis

## Abstract

The study aimed to evaluate whether there is a difference in the expression of programmed cell death 1 ligand (PD-L1) in the cell lining of endometrioma between cases with and without recurrent disease. Additionally, we sought to assess the effect of cyst size and serum CA125 level on the expression of PD-L1 staining. The pathological specimens were immunohistochemically stained for PD-L1 in women who underwent surgery for endometrioma. All patients were evaluated to confirm if their endometriomas had recurred or not. A total of 36 patients who underwent surgery for endometrioma were included. The study population was divided into two groups according to their recurrence status. The study group (having recurrence) (n=12) and the control group (having no recurrence) (n=24) were compared regarding their demographic and clinical characteristics and PD-L1 staining. PD-L1 staining and the intensity of PD-L1 staining did not differ between the patients with and without recurrence. No variable, including parity, cyst size, serum CA125 level, and PD-L1 staining, was found to be significant in determining recurrence. No significant difference was found between the groups with and without PD-L1 staining in terms of cyst size and serum CA125 level. Although we have shown that PD-L1 expression could not be used for the prediction of recurrence, further studies are needed to assess this issue and to guide the development of new immunotherapeutic agents on this basis.

## Introduction

Endometriosis is defined as the presence of endometrial glands and stroma outside the uterine cavity. This estrogen-dependent disorder occurs in approximately 10% of reproductive-aged women, and this rate increases up to 50% among infertile women [1]. Chronic pelvic pain, dysmenorrhea, dyspareunia, and infertility are mostly encountered symptoms in these women. Although the underlying mechanism has not been well understood, the most widely accepted theory is retrograde menstruation as Sampson’s theory. The shed endometrial cells into the peritoneal cavity through the Fallopian tubes have the property of adherence to the peritoneum, proliferation, differentiation, and penetration. Retrograde menstruation is encountered in 70-90% of the women. However, the prevalence of endometriosis is not so high. The reason for this can be explained by the differences in immunologic responses between women. The pathophysiologic process can be understood by the inability of the immunologic system to remove shed endometrial cells as in tumoral development. There is increasing evidence supporting the alterations in both cell-mediated and humoral immunity in the pathogenesis of endometriosis. Increased number and activation of peritoneal macrophages, altered T cell, and natural killer cell cytotoxicities in cellular immunity result in inadequate removal of ectopic endometrial cells from the peritoneal cavity. Another interesting point in the pathogenesis of endometriosis is the inherent resistance of the ectopic endometrial cells against immune cells. It is also associated with the presence of autoantibodies, other autoimmune diseases such as systemic lupus erythematosus, and recurrent abortion. Therefore endometriosis has been considered to be an autoimmune disease [2].

Recurrence of endometriosis is a frequently encountered condition in the postoperative period, which negatively affects the physical and mental health of the patient. Removal of a cyst >8 cm, younger age (<25 years), and preoperative cyst rupture have been proposed as risk factors for recurrence of endometrioma [3]. However, it is not possible to predict the patients in whom endometriomas will recur [4].

Programmed cell death 1 (PD-1) is a transmembrane protein expressed in T cells, B cells, and natural killer cells. It binds to the PD-1 ligand (PD-L1) and PD-L2. PD-L1 is expressed on the surface of multiple tissue types, including many tumor cells and hematopoietic cells. The PD-1 and PD-L1/2 interaction inhibits apoptosis of the tumor cell promoting peripheral T effector cell exhaustion. Based on several Phase II and III trials, antibodies inhibiting PD-1 and PD-L1 have been approved for multiple cancers such as renal cell carcinoma and melanoma [5].

We aimed to evaluate whether there is a difference in the expression of PD-L1 in the cell lining of endometrioma between cases with and without recurrent disease. In addition, we sought to assess the effect of cyst size and serum CA125 level on the expression of PD-L1 staining. We also aimed to identify other variables that would determine the recurrence of endometrioma. This article was previously presented as a meeting abstract at the 4th European Congress on Endometriosis, November 22-24, 2018.

## Materials and methods

**Figure 1:**
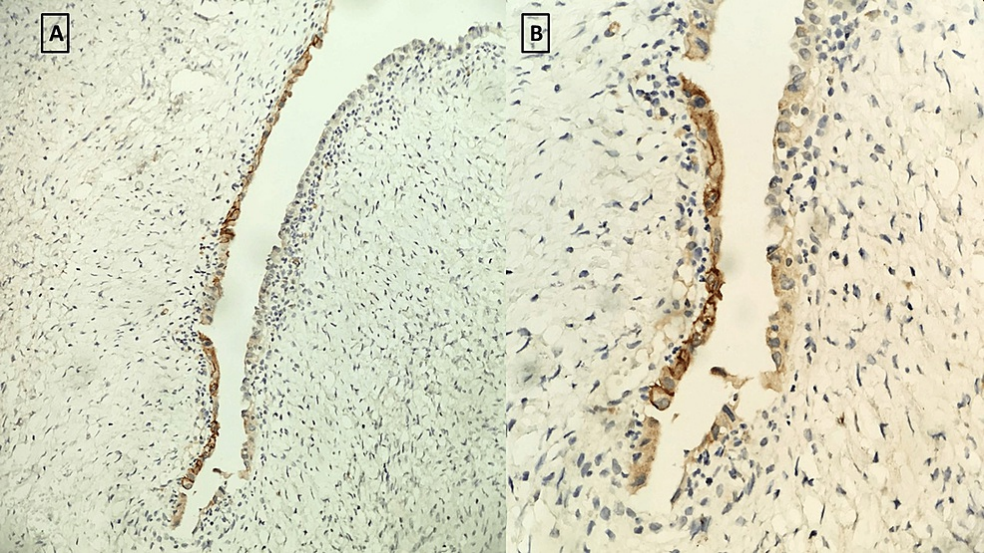
Figure 1A. Endometrial cells in the ovary showing membranous staining with PD-L1 at 10X magnification, DAB chromogen Figure 1B. Endometrial cells in the ovary showing membranous staining with PD-L1 at 40X magnification, DAB chromogen

This retrospective cohort study was conducted at a tertiary referral center. All study participants were selected from patients who underwent surgery for endometriomas. Tissue samples containing endometriosis foci were fixed with 10% neutral buffered formaldehyde, and routine tissue processing was performed. Sections 4-5 µm thick prepared from paraffin-embedded tissue samples were studied immunohistochemically using rabbit monoclonal anti-PD-L1 antibody (clone E1L3N cell signaling) and the Avidin-Biotin-Peroxidase method, with DAB chromogen using a Leica Bondmax automated immunostaining device. Cells showing membranous staining with DAB were counted in areas of endometriosis using routine light microscopy and scored. The intensity of the immunohistochemical staining was scored using a semiquantitative system by an experienced gynecological pathologist. The sections were scored for staining intensity as follows: absent (0), faint (1+), moderate (2+), and intense (3+) (Figure 1). Clinical and demographic characteristics, including age, gravidity, parity, the patient's complaint upon admission, cyst size (cm), serum cancer antigen-125 (CA125) level (IU/L), and surgical characteristics such as the operation route, the presence of adhesion, laterality of the endometrioma, postoperative medication, were obtained from the patients’ medical records. Our hospital’s Ethics Committee (Istanbul, Turkey) approved our study, which was in accordance with the Declaration of Helsinki (diary number 2018-1/2).

All patients were evaluated to confirm if their endometriomas had recurred or not. We divided the study participants into two groups according to their recurrence status. The study group (having recurrence) and the control group (having no recurrence) were compared regarding their demographic and clinical characteristics and PD-L1 staining. Logistic regression analysis was used to determine independent predictors of recurrence in terms of parity, cyst size, serum CA125 level, and PD-L1 staining. The groups were stratified according to mean values of cyst size and serum CA125 level, and a comparison was performed to predict PD-L1 staining.

Statistical analysis was performed with the IBM SPSS Statistics for Windows, Version 22 (Released 2013; IBM Corp., Armonk, New York). Means were presented with standard deviation (SD) and median values for continuous variables. The number of cases and percentages (%) were used for nominal variables. The independent sample t-test and chi-square test were performed. For the multivariate analysis, the possible factors identified with univariate analyses were further entered into the logistic regression analysis to determine independent predictors of recurrence. For all analyses, values of p < 0.005 were considered statistically significant.

## Results

A total of 36 patients who underwent surgery for endometrioma were included in this retrospective cohort study. Table 1 presents the demographic and clinical characteristics of the entire study population. The mean age of the study population was 33.6±7.4 years, while the mean cyst size was 7.6±3.3 cm. Most of the patients (72.2%) were multiparous, and the most common complaint was pelvic pain (58.3%). Laparoscopic cystectomy was performed, and postoperative medication was not recommended in most patients (83.3% and 91.7%, respectively). Of these, PD-L1 staining was detected as positive in the pathological specimens of 19 patients (52.8%).

**Table 1:** Demographic and clinical characteristics of the patients CA125: cancer antigen 125, FSH: follicular stimulating hormone, PD-L1: programmed cell death ligand 1

Characteristics	N(%) or mean ± standard deviation
Age	33.6±7.4
Parity	
Nulliparous	10 (27.8)
Multiparous	26 (72.2)
Cyst size (cm)	7.6±3.3
Serum CA 125 level (U/mL)	301.3±783.3
Serum FSH (IU/L)	7.6±3.6
Serum estradiol (pg/mL)	105.3±135.5
Comorbidity	
Absent	34 (94.4)
Present	2 (5.6)
Complaint	
Only control	7 (19.4)
Pelvic pain	21 (58.3)
Dysmenorrhea/dyspareunia	3 (8.3)
Infertility	5 (13.9)
Previous history of endometriosis	
Absent	31 (86.1)
Present	5 (13.9)
Operation history	
Absent	22 (61.1)
Present	14 (38.9)
Postoperative treatment	
Absent	33 (91.7)
Present	3 (8.3)
Operation	
Laparoscopic cystectomy	30 (83.3)
Cystectomy by laparotomy	3 (8.3)
Oophorectomy	1 (2.8)
Hysterectomy	2 (5.6)
Laterality	
Right	11 (30.6)
Left	16 (44.4)
Bilateral	9 (25)
Adhesion	
Absent	14 (38.9)
Present	22 (61.1)
PD-L1 staining	
Negative	17 (47.2)
Positive	19 (52.8)
PD-L1 staining	
0	17 (47.2)
1	7 (19.4)
2	6 (16.7)
3	6 (16.7)

When the participants in the different groups regarding recurrence were compared, there was no statistical significance between the groups in terms of all demographic and clinical characteristics (Table 2). PD-L1 staining and the intensity of PD-L1 staining also did not differ between the patients with and without recurrence.

**Table 2:** Comparison of the patients with and without recurrence regarding their demographic and clinical characteristics *Independent sample t-test and chi-square test were applied; p<0.05 was considered statistically significant CA125: cancer antigen 125, FSH: follicular stimulating hormone, PD-L1: programmed cell death ligand 1

Characteristics	N(%) or mean ± standard deviation		p-value*
	Group 1 (no recurrence, n=24)	Group 2 (recurrence, n=12)	
Age	33.0±7.7	34.6±6.9	0.561
Parity			
Nulliparous	7 (29.2)	3 (25)	0.792
Multiparous	17 (70.8)	9 (75)	
Cyst size (cm)	7.8±3.6	7.3±2.7	0.715
Serum CA125 level (U/mL)	331.7±900.9	240.3±499.0	0.747
Serum FSH (IU/L)	9.3±4.3	6.3±2.8	0.298
Serum Estradiol (pg/mL)	153.7±213.5	69.0±47.4	0.565
Comorbidity			
Absent	22 (91.7)	12 (100)	0.303
Present	2 (8.3)	0	
Complaint			
Only control	5 (20.8)	2 (16.7)	0.877
Pelvic pain	13 (54.2)	8 (66.7)	
Dysmenorrhea/dyspareunia	2 (8.3)	1 (8.3)	
Infertility	4 (16.5)	1 (8.3)	
Operation history			
Absent	15 (62.5)	7 (58.3)	0.809
Present	9 (37.5)	5 (41.7)	
Postoperative treatment			
Absent	22 (91.7)	11 (91.7)	1.000
Present	2 (8.3)	1 (8.3)	
Operation			
Laparoscopic cystectomy	20 (83.3)	10 (83.3)	0.861
Cystectomy by laparotomy	2 (8.3)	1 (8.3)	
Oopherectomy	1 (4.2)	0	
Hysterectomy	1 (4.2)	1 (8.3)	
Laterality			
Right	10 (41.7)	1 (8.3)	0.123
Left	9 (37.5)	7 (58.3)	
Bilateral	5 (20.8)	4 (33.3)	
Adhesion			
Absent	9 (37.5)	5 (41.7)	0.809
Present	15 (62.5)	7 (58.3)	
PD-L1 staining			
Negative	10 (41.7)	7 (58.3)	0.345
Positive	14 (58.3)	5 (41.7)	
PD-L1 staining			
0	10 (41.7)	7 (58.3)	0.731
1	5 (20.8)	2 (16.7)	
2	5 (20.8)	1 (8.3)	
3	4 (16.7)	2 (16.7)	

No variable, including parity, cyst size, serum CA125 level, and PD-L1 staining was found to be significant in determining recurrence according to logistic regression analysis as shown in Table 3.

**Table 3:** Evaluation of the effect of variables on recurrence by logistic regression analysis *Multiple linear regression analysis was applied, p<0.05 was considered statistically significant OR: odds ratio, CI: confidence interval, CA125: cancer antigen 125, PD-L1: programmed cell death ligand 1

Variables	OR	p-value^*^	95% CI	
Parity group	1.43	0.686	0.25	8.16
Cyst size (mm)	1.92	0.428	0.38	9.59
Serum CA125 level (IU/L)	1.68	0.612	0.23	12.62
PD-L1 staining	0.46	0.297	0.11	1.98

Table 4 presents the comparison of the patients with and without PD-L1 staining in terms of cyst size and serum CA125 level. No significant difference was found between the groups (p = 0.429 and p = 0.727).

**Table 4:** Comparison of the patients with and without PD-L1 staining in terms of cyst size and serum CA125 level *Chi-square test was applied; p < 0.05 was considered statistically significant CA125: cancer antigen 125, PD-L1: programmed cell death ligand 1

Characteristics	N (%) or mean±standard deviation		p-value*
	PD-L1 staining (-), (n=17)	PD-L1 staining (+), (n=19)	
Cyst size (mm)			
<7.6 mm	12 (70.6)	11 (57.9)	0.429
≥7.6 mm	5 (29.4)	8 (42.1)	
Serum CA125 level (IU/mL)			
<300 IU/mL	15 (88.2)	16 (84.2)	0.727
≥300 IU/mL	2 (11.8)	3 (15.8)	

## Discussion

Our aim in this retrospective cohort study was to detect whether there is any difference in PD-L1 staining of endometrioma between cases with and without recurrent disease and to determine whether it can be used as a marker for the prediction of endometrioma recurrence. We also investigated whether cyst size and serum CA125 level would affect PD-L1 staining. The key finding of our study was that PD-L1 staining did not show any significant difference in patients with recurrent disease, and so it cannot be used as a marker to predict recurrence. Cyst size and serum CA125 level did not remain significant determinants for PD-L1 staining. Additionally, recurrence was not significantly affected by any other demographic and clinical characteristics of the patients.

Programmed cell death-1, expressed on the surface of activated T cells, B cells, and other lymphocytes, binds specifically with PD-L1. The programmed cell death-1 ligand has an immunosuppressive role through the inhibition of T-cell activity [6]. The interaction between PD-1 and PD-L1 is important for immune responses and peripheral tolerance. Immune system disorders develop because of the suppression of protective T-cell responses under pathological conditions depending on the abnormality of this PD-1 and PD-L1 pathway. Thus, the expression of PD-1 and PD-L1 has been shown in several autoimmune diseases and cancers such as atherosclerosis, periodontitis, colorectal cancer, renal cell carcinoma, and melanoma [7-9]. Immunotherapeutic agents targeting the PD-1/PD-L1 pathway have been developed as a novel treatment option for these diseases [10, 11]. New promising therapeutic approaches for several malignancies, including non-small cell lung carcinoma and melanoma, have been developed based on this underlying relationship [12].

Endometriosis is defined as the condition where tissue similar to the lining of the womb grows in other places, such as the ovaries and the Fallopian tubes. This disease is an inflammatory, estrogen-dependent condition associated with pelvic pain and infertility. Several pathophysiologic mechanisms are responsible for the development of endometriotic lesions, including retrograde menstruation, coelomic metaplasia, lymphovascular metastasis, abnormal angiogenesis, dysregulation of apoptotic mechanisms, oxidative stress, immune dysfunction, and stem cell theory. There are a limited number of papers in the literature investigating the involvement of the PD-1/PD-L1 pathway in endometriosis patients, as immune dysregulation plays an important role in the development and promotion of endometriosis, in addition to endocrine dysregulation [13, 14]. Elevated PD-1/PD-L1 expression in both eutopic and ectopic endometria and peripheral blood cells has been detected in women with endometriosis [15]. It has been suggested that the detection of PD-1 and PD-L1 on T and B cells would be an indicator of an impaired immune system in women with endometriosis. Deregulation of the PD-1/PD-L1 pathway has even been associated with a poorer prognosis in terms of response to treatment [16]. Treatment with 17beta-estradiol has been shown to upregulate PD-L1 expression in eutopic epithelial cells [17].

Recurrence following endometriosis surgery is an important issue that should be prevented to increase the quality of life. Endometrioma recurrence may be experienced in approximately 25% of patients who undergo surgical removal of endometrioma [18]. Hormonal suppression may be considered for these patients having no wish for pregnancy immediately after surgery in order to reduce disease recurrence and pain. If we develop a biomarker that predicts recurrence, the management of these patients will be more accurate and individualized according to each woman’s needs.

The retrospective design and small sample size could be accepted as the limitations of the study. However, to the best of our knowledge, this is the first study assessing the association between endometriosis recurrence and PD-L1 expression. We found no significant association, and PD-L1 expression could not be a good predictor of endometriosis recurrence.

Another important issue is the development of endometriosis-associated ovarian cancer. When Nero et al. evaluated whether the presence of PD-1/PD-L1 expression is a predisposing factor for the development of endometriosis-associated ovarian cancer, higher PD-1 levels were encountered in these cases. It has been suggested that benign cases with high PD-1/PD-L1 expression may also develop cancer at an earlier period [19]. The combination of PD-1/PD-L1 blockade with immunotherapies in endometriosis and/or endometriosis-associated ovarian cancer may improve treatment responses [20].

Since PD-L1 expression does not show a significant difference in endometriosis recurrence, we did not consider the usability of antibodies against PD-L1 for the management of endometrioma. However, further prospective studies in larger populations are needed to understand whether the treatment modality to be developed on this basis has a role in preventing recurrence. In addition, different PD-L1 clones may be required depending on the disease being treated if a clone other than the standardized one is used.

## Conclusions

It is known that changes in PD-L1 expression play an important role in the development of endometriosis, as immunological dysfunction is one of the pathophysiologic mechanisms responsible for its development and promotion. Although our study has shown that PD-L1 expression could not be used for the prediction of recurrence, further research is necessary to elucidate this relationship and to guide the development of new immunotherapeutic agents based on these findings.
